# Investigation of N-(2-oxo-2H-chromen-3-carbonyl)cytisine’s Crystal Structure and Optical Properties

**DOI:** 10.3390/ma18133153

**Published:** 2025-07-03

**Authors:** Anarkul Kishkentayeva, Kymbat Kopbalina, Zhanar Shaimerdenova, Elvira Shults, Yury Gatilov, Dmitrii Pankin, Mikhail Smirnov, Anastasia Povolotckaia, Dastan Turdybekov, Nurlan Mazhenov

**Affiliations:** 1 School of Pharmacy, Karaganda Medical University, Karaganda 100012, Kazakhstan; anar_kish@mail.ru (A.K.); arsenzhan@bk.ru (Z.S.); 2 Department of Physics and Nanotechnology, Buketov Karaganda University, Universitetskaya 28, Karaganda 100024, Kazakhstan; 3 N.N. Vorozhtsov Novosibirsk Institute of Organic Chemistry, Siberian Branch of the Russian Academy of Sciences, 630090 Novosibirsk, Russia; schultz@nioch.nsc.ru (E.S.); gatilov@nioch.nsc.ru (Y.G.); 4 Center for Optical and Laser Materials Research, St. Petersburg State University, Ulianovskaya 5, 198504 St. Petersburg, Russia; anastasia.povolotckaia@spbu.ru; 5 Faculty of Physics, St. Petersburg State University, Universitetskaya Nab. 7/9, 199034 St. Petersburg, Russia; m.smirnov@spbu.ru; 6 Department of Physics, Abylkas Saginov Karaganda Technical University, Nazarbayev 56, Karaganda 100027, Kazakhstan; turdas@mail.ru (D.T.); mazhenov@mail.ru (N.M.)

**Keywords:** coumarin, cytisine, FTIR, XRD, DFT calculations, phonons, crystal, vibrational modes, electronic band structure

## Abstract

Coumarin and cytisine and their derivatives have significant biological activity. In addition, the electronic properties of coumarin derivatives are very sensitive to the molecular environment, which allows for their use as sensors for bioluminescent imaging. Due to the fact that cytisine exhibits high activity in binding to nicotinic acetylcholine receptors, a compound combining parts of cytisine and coumarin may have a broader spectrum of biological activity and also act as a photoactive element for promising use in optoelectronic devices. This article reports the synthesis of a crystalline cytisine–coumarin complex (IUPAC: N-(2-oxo-2H-chromene-3-carbonyl)cytisine), along with the results of both theoretical and experimental investigations of its structural and electronic properties. The structure of this new compound was established on the basis of X-ray diffraction and Fourier transform infrared spectroscopy data and was confirmed through density functional theory calculations using periodic crystal and single-molecule approaches. Interpretations of the IR absorption peaks and the atomic patterns of the vibrational modes are given. The electronic band structure and the contributions of individual atoms to the electronic density of states are analyzed. The structural and optical properties considered may be useful for quality control of the compound and for studying similar matrices.

## 1. Introduction

The development of simple and effective methods for the synthesis of new compounds based on cytisine and coumarin is a promising pharmacological challenge. This is due to the high biological activity of derivatives of cytisine and coumarin compounds. Thus, among others, antimicrobial [1,2,3], anti-inflammatory [1,2], antidiabetic [2,4,5], anticancer [1,2,6,7], antitumor [6], and anti-proliferative [6] activities, as well as anticoagulant [2,5] biological effects, of coumarin and its derivatives have been noted in a number of previous studies. Moreover, as substances with a natural origin that can be synthesized, coumarins have minimal side effects. In addition to their pronounced biological properties, coumarins and their derivatives have intense luminescence, with sensitivity to the environment and the preparation conditions [5], making their derivatives promising for various applications in photoelectronic devices and sensors. In particular, a number of studies have proposed coumarin derivatives as luminescent probes [8,9] for bioimaging tasks [10,11] and as sensors for specific compounds [12,13] and elements [14,15].

Cytisine, as a natural alkaloid, also exhibits a number of important biological properties. Its significant ability to bind to neuronal nicotinic acetylcholine receptors (nAChRs) [16] has played an important role in both the development of drugs that reduce smoking cravings [17,18] and in fundamental studies on the functioning of the central nervous system [16]. Studies conducted on selected lines of rats bred at the University of Chile exhibiting alcohol drinking behavior (UChB) have also demonstrated the promise of cytisine in reducing alcohol cravings [19]. Other promising areas of research into cytisine and its derivatives involve their use against depression, Alzheimer’s disease, Parkinson’s disease, arrhythmia, and epilepsy [17,20,21].

Currently, the molecular hybridization approach demonstrates potential for synthesizing future drugs for pharmacology [22,23]. Particular attention has been paid to coumarin-based compounds [22,23]. In these studies, the synthesis of new cytisine–coumarin compounds, combining the advantages of both compounds, is expected to expand the capabilities of the current drugs and contribute to the development of phototherapy methods. In order to demonstrate the potential properties of a synthesized molecule, quality control of the grown structure of the compound is important. This will subsequently open up the possibilities for more accurate studies of biological activity and biocompatibility. Studies on the synthesis of cytisine derivatives of coumarins can be found in Refs. [24,25]. The structure of such compounds and the location of their specific sites determine their chemical and biological activity. An understanding of the electronic structure of these compounds will contribute to further practical applications, both in the field of pharmacology, with switching of their biological activities [21,26,27], and in the creation of field sensors for the visualization of the distribution of medicinal substances [10,11,12,13]. In addition, the possibility of using spectral methods based on reliably established structure–spectrum correlations is important for controlling the structure of the compounds obtained during production at the industrial scale.

Modern quantum chemical methods provide significant information for the study of the structures of new compounds and their interactions with the environment. The density functional theory (DFT) approach makes it possible to predict the structural and optoelectronic properties of studied compounds with fairly good accuracy and relatively low computational costs. Depending on the type of aggregate state, a distinction is made between crystal calculations (calculations using periodic boundary conditions) and calculations for a single molecule in solution (or in the gas phase, with the properties scaled to account for the condensation effect). Due to the interest in studying the structural and optical properties of a compound in solution, a single-molecule calculation approach corrected for environmental effects is often used [5,28,29]. It should be noted that the structure of compounds in solution may differ from that in crystal form due to the flexibility of the molecular framework and the influence of the solvent. A set of different conformational states may be present in solution, which should be taken into account [30]. Theoretical studies of compounds’ crystal structures are performed much less often. This may be due to the higher computational costs incurred when the unit cell contains a huge number of molecules and there is relatively low symmetry in the molecular crystal. There may also be experimental difficulties associated with growing a single crystal. Nevertheless, it should be noted that the information obtained during modeling and experimental research makes it possible to more accurately understand the nature of the ordering of the molecules and hydrogen bonding in a crystal, as well as the structure of the electronic bands. The crystalline case is more consistent with the drug release for tablets, which means that studying crystals allows us to develop methods for the quality control of manufactured pharmaceutical products. Moreover, quality control of synthesized compounds is closely related to the quality of the operation of devices based on these compounds due to their special optoelectronic properties. For compounds in a crystalline state, their structures are often investigated using X-ray diffraction (XRD) and vibrational spectroscopy techniques. Among the latter, the Fourier transform infrared spectroscopy (FTIR) method, unlike Raman spectroscopy, where luminescence can produce significant interference, allows for easier quality control of the synthesized compounds from the point of view of their implementation. Additional advantages when implementing the FTIR reflectance spectroscopy technique or the diffuse reflectance infrared Fourier transform spectroscopy (DRIFTS) technique are also lack of contact with and the indestructibility of the sample.

In this regard, the aim of this study was

- To synthesize a cytisinyl–coumarin complex compound (hereafter, the word “complex” is used in order to highlight that the investigated molecule is made up of a cytisine moiety and a coumarin moiety);
- To characterize its structure through XRD and FTIR spectroscopy methods;
- To study its structural, vibrational, and electronic properties using DFT computational modelling.

This theoretical study was carried out within the framework of both periodic crystal and single-molecule approaches. The information obtained will be useful in further characterizing similar compounds and establishing the structure–spectrum correlations used in practice for quality control.

## 2. Materials and Methods

### 2.1. Synthesis

Synthesis of the cytisinyl–coumarin complex (N-(2-oxo-2H-chromen-3-carbonyl)cytisine) was performed using coumarin acid chloride. The production of coumarin acid chloride was carried out in several stages.

At the first stage, synthesis of 2-oxo-2H-chromene-3-carboxylic acid (сoumarin-3-carboxylic acid) from 2-hydroxybenzaldehyde (salicylaldehyde, CAS Number 90-02-8, 98% purity, Merck, Darmstadt, Germany) and 2,2-Dimethyl-1,3-dioxane-4,6-dione (Meldrum’s acid, CAS Number 2033-24-1, 98% purity, Merck, Darmstadt, Germany) was carried out according to Ref. [31]. At the second stage, coumarin acid chloride (**1**) was obtained by reacting Coumarin-3-carboxylic Acid with SOCl_2_, as described in [30].

**Figure 1:**
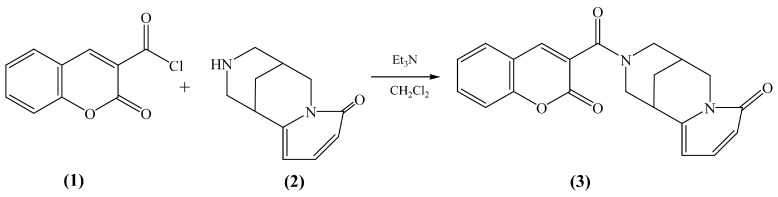
Final stage of cytisine–coumarin (**3**) synthesis from coumarin chloride (**1**) and cytisine alkaloid (**2**).

The cytisinyl-coumarin complex (**3**) (Figure 1) was obtained as follows: 4.8 mmol of cytisine alkaloid ((-)-cytisine, CAS Number 485-35-8, 99% purity, enantiopure, Merck, Darmstadt, Germany) (**2**) and 24 mmol of triethylamine were added to a solution of 4.8 mmol of coumarin acid chloride (**1**) in 35 mL of methylene chloride under stirring. The reaction was carried out at room temperature for 5 h. The organic layer was treated 3 times in a separation funnel with a solution of methylene chloride mixed with water at a 1:1 ratio. The growth was performed layer by layer. The organic layer was dried over magnesium sulfate for two hours and then filtered and evaporated in a rotary evaporator. Then, column chromatography on silica gel was carried out. As a result, a powdery substance of crystals was isolated.

The cytisinyl–coumarin complex (**3**) (hereafter simply referred to as the “complex”) was obtained for the first time. No matches were found when comparing it with the structures from the Cambridge database. The structure of compound (**3**) was established through single-crystal X-ray diffraction, with the experimental results discussed in Section 2.2 and the theoretical modeling discussed in Section 2.3. The melting point (MP) was determined to be 237 °C (fracture). The details of the experiment are described in Section 2.2. The obtained structure is presented in the corresponding CIF file in the Supporting Materials and was deposited on the Cambridge Crystallographic Data Centre (CCDC) site. The CCDC deposition number is 2393757.

### 2.2. The Experimental Approach

Control over the course of the chemical transformations and the purity of the products obtained during this study was carried out by thin-layer chromatography (TLC) on chromatographic plates “Sorbfil” PTSX-P-A-UV (Imid, Krasnodar, Russia) using polyethylene terephthalate substrate material, 254 nm of phosphor, and a plate size of 10 cm × 15 cm (Sorbpolymer, Krasnodar, Russia) in the chloroform–ethanol solvent system (a 10:1 ratio). Spot detection was carried out using a UFS-254/365 irradiator (Imid, Krasnodar, Russia).

The reaction product was purified using column chromatography and crystallization from various organic solvents (ethyl alcohol, chloroform). The columns were filled with silica gel L 100/160 µm.

The melting point was determined using the OptiMelt MPA100 device in automatic mode (Stanford Research Systems, Sunnyvale, CA, USA).

IR spectra were obtained using the Avatar 360 ESP FTIR spectrometer (Thermo Nicolet, Madison, WI, USA) in the 1000–4000 cm^−1^ spectral region. The spectral resolution was 4 cm^−1^. The number of scans was 100 items. The detector was deuterated triglycine sulfate (DTGS), and the light source was a Globar (SiC) lamp. A KBr beamsplitter was used. The spectra were obtained from the pellet in transmittance mode. In order to obtain the pellet, 1.2 mg of the sample was pressed using 300 mg of KBr. The former was preliminarily heated at 150 °C for 3 h.

The single-crystal X-ray diffraction experiment was performed on an APEX-II diffractometer (Bruker, Berlin, Germany) equipped with a charge-coupled device (CCD) detector. It was equipped with a graphite monochromator, λ(Mo-Kα) = 0.71073 Å. The temperature was 297 К, and a φ,ω-scan was performed. The absorption was accounted for using the empirical method in the SADABS 2016/2 program [32]. The structure of the compound was deciphered using the direct method according to the SHELXT-2014 [33] program and refined within the anisotropic–isotropic (for H atoms) approximation according to the SHELXL-2018 program [34]. The positions of the hydrogens were calculated geometrically, and the parameters of the hydrogen atoms were refined within the isotropic approximation in the “rider” model. The analysis of the molecular geometry and intermolecular interactions was carried out using PLATON [35].

### 2.3. The Theoretical Approach

The calculations were performed within the generalized gradient approximation (GGA) of the density functional theory with the Perdew–Burke–Ernzerhoff (PBE) functional [36], taking into account the dispersion correction using the Tkatchenko–Scheffler (TS) method [37]. It will hereafter be called the TS-GGA-PBE approach. The norm-conserving pseudopotentials implemented were used in the Castep program 7.0 [36,38]. A plane-wave basis set with a cutoff energy of 1000 eV was used. The convergence criterion in the self-consistent field procedure was assumed to be 5 × 10^−7^ eV/atom. The modified Broyden–Fletcher–Goldfarb–Schanno (LBFGS) method was used to optimize the geometry [39] until the residual stresses, residual forces, and maximum displacements were smaller than 0.02 GPa, 0.01 eV/Ȧ, and 5 × 10^−4^ Ȧ, respectively. The dimensions of the Monkhorst–Pack grid were chosen to provide an overall k-vector step equal to 0.04 1/Ȧ. Calculation of the vibrational frequencies, as well as the IR absorption intensities, was carried out using the linear response method from the perturbation theory [40,41]. The results obtained were compared with the experimental IR absorbance, the advantages and disadvantages of the theoretical approach have been discussed. This information can be useful not only for modeling the vibrational properties of the given compound but also in providing general information about the applicability of such an approach. Currently, there are information and benchmarks about the lattice parameter modeling and phase transitions using this approach [42,43]. However, there is very limited information about modeling the electronic [44] and vibrational properties within the TS-GGA approach. An energy range of 20 eV was chosen to calculate the band structure with k-point separation of 0.015 1/ Ȧ. The standard setting of the Brillouin zone path was selected, supplemented by the paths defined in Ref. [45]. Due to the fact that the TS-GGA-PBE approach tends to underestimate the band gap value, the optical absorption spectrum for the polycrystalline case was corrected using the scissor operator. For this purpose, the calculated spectrum was compared with the results of the absorption spectrum calculated (singlet–singlet transitions) for a single-molecule approach, as well as with the experimental UV-Vis absorption spectrum (Figure S1). As a result, the scissor operator with an empirical value equal to 0.82 eV was applied. To compare the results of the solid-state calculation, a calculation was also performed within the framework of density functional theory for a single molecule in the gas phase using the Gaussian G09W Rev. C.01 software (Gaussian Inc, Wallingford, CT, USA) [46]. The Becke three-parameter Lee–Yang–Parr (B3LYP) functional with the 6-311G(2d,p) basis set was used [47,48]. Hereafter, this approach will be referred to as the B3LYP approach. Using this approach in previous studies, the structural and optical properties could be accurately reproduced [49,50,51]. Optimization was performed until the standard criteria for the maximum and RMS residual force and displacement convergence were met. The stability of the optimized structures was confirmed by the absence of the imaginary vibrational frequencies. The influence of intermolecular interactions on the frequencies of the vibrational modes was taken into account using a scaling procedure. Based on previous calculations using a similar approach [49,50] and the experimentally determined positions of spectral peaks, the scaling factor was chosen to be 0.98. For practical reasons, the modes in the 400–4000 cm^−1^ region are discussed in the corresponding section. Visualization of the obtained results was performed using the GaussView software ver. 5.0.9 (Gaussian Inc, Wallingford, CT, USA) and the Origin 9.0 software (OriginLab Co.; Northampton, MA, USA). In some cases where the results of the two theoretical approaches are qualitatively similar, the results of the single-molecule approach are demonstrated.

## 3. Results and Discussion

### 3.1. Structural Properties

#### 3.1.1. The Stability Test

In accordance with the method described in Section 2.1, synthesis of the investigated compound was carried out. The initial investigation of the crystal structure was performed using the XRD technique. It was found that at room temperature, the crystal system with two molecules in the unit cell and the space group P2_1_ (#4) is monoclinic. The experimental unit cell parameters are given in Table 1.

This experimental crystal structure was used as the starting point for geometry optimization, and subsequent calculation of the vibrational frequencies at the Г point led to the detection of one vibrational mode with the imaginary frequency (17.71 i cm^−1^). The atomic displacements in this vibrational mode are demonstrated at Figure S2. The most significant atomic displacements in this mode are localized mainly in the cytisine part. After distorting the molecular structure along the eigenvector of this mode (as shown in Figure S2), the structure was again optimized. The calculated vibrational spectrum of the obtained crystal structure with the space group P1 confirmed its stability. The theoretically optimized unit cell parameters are also given in Table 1. As can be seen, the angles α and γ are very close to 90°, and the calculated β is close to the experimental value. The largest deviation in the unit cell vectors is noted for the *b* length (about 2.8%). It should be noted that the calculation was carried out at 0 K, and the XRD measurements were carried out at room temperature (*T* = 296 ± 2 K). In this connection, one cannot exclude the possibility of an ordered–disorder phase transition from the P1 phase to the more symmetric P2_1_ phase at an increasing temperature. The calculation predicts the total energy corrected for the finite basis set as −11,606.31903 eV for the stable state (P1 symmetry) and −11,606.31264 eV for the transition state with the imaginary vibrational frequency (P2_1_ symmetry). Thus, the predicted energy barrier is 6.4 meV. This value is much smaller than the thermal energy at room temperature *k_B_T* = 25.5 meV.

**Figure 2:**
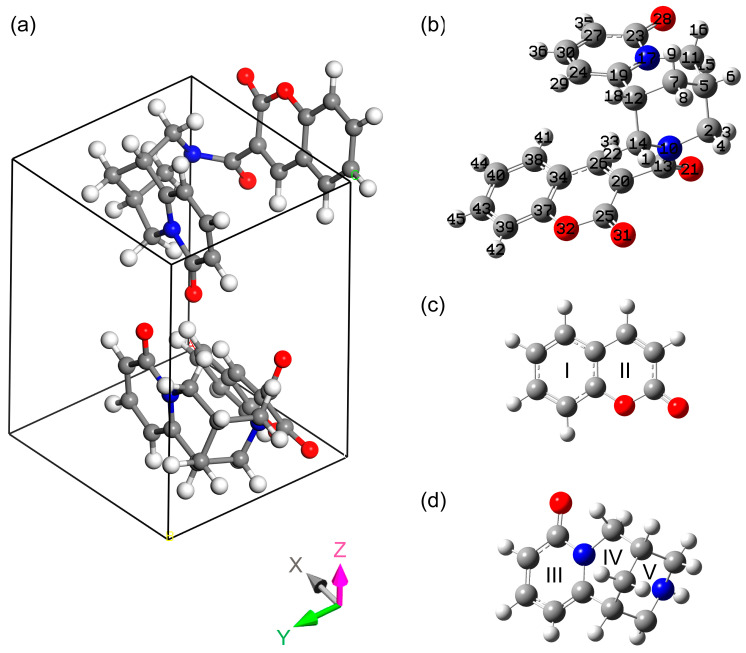
The optimized structure of the crystal unit cell (**a**); the optimized geometry of a single molecule in the gas phase (**b**) (hereinafter, this atom numbering is used in the text; similar atom numbering is used in the CIF file in the Supporting Materials); cytisine (**c**) and coumarin (**d**) molecular structures with ring numbering. Hereafter, carbon, oxygen, nitrogen and hydrogen atoms are dark gray, red, blue, and light gray, respectively.

Subsequently, we will consider a stable crystal structure with P1 symmetry (hereafter, simply “crystal”). Due to the formal difference in the molecular structures of the two molecules in the unit cell, the parameters for both molecules are given in Table 2. Since the complex molecule shares similar structural features to the coumarin and cytisine molecules (see Figure 2), we will compare the complex molecules with these individual molecules in the following sections.

#### 3.1.2. The Coumarin Moiety

The experimentally determined structural parameters of the molecular complex were compared with the corresponding structural parameters of the coumarin and cytisine molecules defined in [52] and [53], respectively (see Table 2 and Table S1). There are no significant differences between the complex molecule and free coumarin from the literature [52]. In the case of the C40-C43 bond, the intermolecular interaction between the hydrogen atom H44 in one molecule and the oxygen atom O31 in another is presented, and the distance between them is 2.595(3) Å. In the case of the C26-C34 bond, the shorter CC distance in the complex is presumably due to the influence of the cytisine moiety and the intermolecular interaction with the hydrogen atoms from the other molecules (H41 and H33). The length of the O28-H41 and O28-H33 hydrogen bonds is 2.474 and 2.340 Å, respectively. The ratios between the bond lengths in the complex are qualitatively similar to those in the coumarin molecule. So, the shortest bond in this complex moiety is C25=O31. The shortest of the double carbon bonds is C20=C26. For the other double carbon bonds, the experimental lengths range within 1.371–1.397 Å.

In the coumarin moiety, the C25-C20 single bond has the longest length, while the C37-O32 and O32-C25 single bonds have experimental lengths of 1.385 and 1.375 Å.

The theoretical calculations for the stable state both in the crystalline and single-molecule approximations are qualitatively consistent with the structural identification on the basis of the XRD results. It should be noted that there is a systematic overestimation of the lengths of the double carbon bonds in the calculations, which is especially evident for ring I. Thus, the greatest discrepancy is noted for С40-С43 (in ring I), C2-C26 (in ring II), and C34-C37 (at the boundary of rings I and II); see Figure 2 and Table 2. At the same time, the discrepancies in the lengths of the double bonds between the calculation and the experiment are smaller for the single-molecule approach, which was also noted in a number of other works using similar methods [49,50].

The plane angles around the C40 atom in ring I of the complex are closer to 120° (see Table S1). A greater deviation is noticed for the plane angles around the C37 atom. Similar tendencies in the plane angles between the coumarin molecule and the complex molecule can be noted. This is also reproduced by the calculations for both approximations.

The values for the dihedral angles in the polycyclic coumarin moiety are very small (approximately 1.5° or about 180°) in both the experiment and the calculation. The dihedral angles at the cytisine and coumarin moiety boundary are much larger, as is well reproduced in the crystalline approximation (see Table S1). On the other hand, the discrepancies between the experiment and the calculation for a single molecule demonstrate possible structural flexibility and a tendency towards rotational conformers.

**Table 2:** Selected bond lengths of the coumarin and cytisine moieties according to the experimental data and theoretical predictions.

Bond Length, Å	Experimental Data for Complex Crystal SG P2_1_ (This Work)	Experimental Data for Coumarin Crystals [54,55] and Cytisine Crystals [53] *	Theoretical Data for Complex Crystal Approach, SG P1 Molecule1 (Molecule2)	Theoretical Data for Single-Molecule Approach
**Coumarin moiety**				
C34-C38	1.397(4)	1.404(3)	1.410 (1.411)	1.404
C38-C40	1.371(7)	1.375(3)	1.387 (1.387)	1.381
C40-C43	1.371(7)	1.397(4)	1.405 (1.406)	1.399
C43-C39	1.384(6)	1.380(4)	1.393 (1.392)	1.385
C39-C37	1.379(6)	1.383(3)	1.396 (1.396)	1.390
C20-C26	1.337(4)	1.347(4)	1.364 (1.365)	1.352
C26-C34	1.426(5)	1.434(3)	1.428 (1.428)	1.432
C34-C37	1.384(4)	1.399(3)	1.408 (1.408)	1.402
C37-O32	1.385(4)	1.384(3)	1.371 (1.371)	1.362
O32-C25	1.375(4)	1.374(3)	1.397 (1.397)	1.391
C25-C20	1.451(4)	1.448(3)	1.452 (1.453)	1.463
C20-C13	1.506(5)	--	1.509 (1.512)	1.510
C13-O21	1.231(4)	--	1.239 (1.239)	1.223
C25-O31	1.204(4)	1.214(3)	1.220 (1.220)	1.203
**Cytisine moiety**				
N17-C23	1.403(5)	1.405(6)	1.432 (1.433)	1.427
C23-C27	1.415(8)	1.424(7)	1.436 (1.435)	1.441
C27-C30	1.328(9)	1.381(8)	1.372 (1.372)	1.359
C30-C24	1.386(6)	1.399(8)	1.412 (1.412)	1.413
C24-C19	1.355(6)	1.357(7)	1.378 (1.378)	1.368
C5-C11	1.503(5)	1.530(7)	1.530 (1.528)	1.531
C11-N17	1.473(6)	1.486(6)	1.486 (1.484)	1.480
N17-C19	1.365(4)	1.388(5)	1.377 (1.376)	1.369
C19-C12	1.495(5)	1.501(7)	1.512 (1.513)	1.512
C12-C7	1.514(6)	1.528(8)	1.531 (1.531)	1.530
C2-C5	1.523(5)	1.533(7)	1.538 (1.539)	1.535
C5-C7	1.517(6)	1.538(7)	1.530 (1.530)	1.530
C7-C12	1.514(6)	1.528(8)	1.531 (1.531)	1.530
C12-C14	1.529(5)	1.541(7)	1.545 (1.544)	1.549
C14-N10	1.449(4)	1.463(6)	1.465 (1.466)	1.454
N10-C2	1.460(5)	1.468(6)	1.468 (1.472)	1.461
N10-C13	1.338(4)	--	1.370 (1.371)	1.365

* For the experimental data, the uncertainties are given in brackets.

#### 3.1.3. The Cytisine Moiety

A comparison of the experimental structural parameters for the complex molecule and a single cytisine molecule shows that the greatest difference in the bond length takes place for ring III in the cytisine moiety, namely for the shortest C27=C30 bond in it (see Table 2). This may be due to the interaction of the coumarin and cytisine double carbon bonds inside the molecule through the π orbitals. The shortest ones in the cytisine moiety are the double carbon bonds C27=C30 and C24=C19, as well as the carbon–nitrogen bond C19-N17 at the boundary of the III and IV rings. It can be also noted that according to the XRD data, the lengths of the single carbon–carbon bonds in rings IV and V exceed 1.495 Å. The carbon–nitrogen bond lengths in rings III, IV, and V in turn range from 1.403 to 1.460 Å. By analogy with the coumarin moiety, the TS-GGA-PBE approach mainly overestimates these bond lengths. The greatest absolute overestimations are noted for ring III with the double bonds and especially for the C27=C30 bond. However, the main relations determined experimentally between different bond lengths are well reproduced by the calculations (see Table 2).

Comparing the calculated plane angles with the experimental data, it is possible to note coincidence within 1° (see Table S1). A comparison of the experimental values of the complex and a single molecule from Ref. [53] shows that the greatest differences are localized at the boundary between the IV and V rings at angles C19C12C7 and C7C5C11, as well as near the boundary between the V ring and the transition region. For angles C14N10C2 and N10C2C5, the differences are about 2°.

Rings IV and V do not contain multiple bonds and thus are flexible. The dihedral angles in these molecular fragments are rather scattered. In Table 3, they are compared with those reported in Ref. [53]. It can be noted that ring IV is significantly curved relative to the plane of ring III. This manifests itself in the values for the C23N17C11C5 and C24C19C12C7 boundary dihedral angles. The greatest absolute changes in the dihedral angle inside ring IV concern angles C5C11N17C19 and C7C5C11N17 (see Table S1). Also, the structural flexibility of rings IV and V is consistent with the large deviations in the dihedral angle values calculated using the single-molecule approach. As for the coumarin moiety, in the case of the cytisine moiety, the B3LYP/6-311G(2d,p) method predicts the bond lengths more accurately, especially for the double bonds.

While considering the cytisine moiety, it should also be noted that it contains a number of the shortest intramolecular hydrogen bonds of the type C=O---H (see Table 4). They are formed due to the rotation of one of the methylene group in rings IV and V towards the oxygen atom in the C=O unit. These are, for example, H3-O21 and H15-O28 contacts. It should be noted that in addition to these, there is also a longer hydrogen bond through O31 of the coumarin moiety and the methylene group of the cytisine moiety, but their characteristic lengths are much longer: 2.755 and 3.155 Å for the H22-O31 and H1-O31 contacts, respectively.

The above-mentioned difference in the dihedral angles calculated using the crystalline and molecular approaches is due to accounting or not accounting for the intermolecular interactions. This aspect is closely related to the various descriptions of the hydrogen bonds (see Table 3).

### 3.2. Vibrational Properties

One experimental method sensitive to the structural features is vibrational spectroscopy, namely FTIR absorption spectroscopy in the mid-IR region. In order to use spectroscopic information, it is important to establish the structure–spectrum relation. Such relations provide us with valuable information for understanding the structural peculiarities of the system under study and allows us to conclude how reliable the applied calculation methods are.

**Figure 3:**
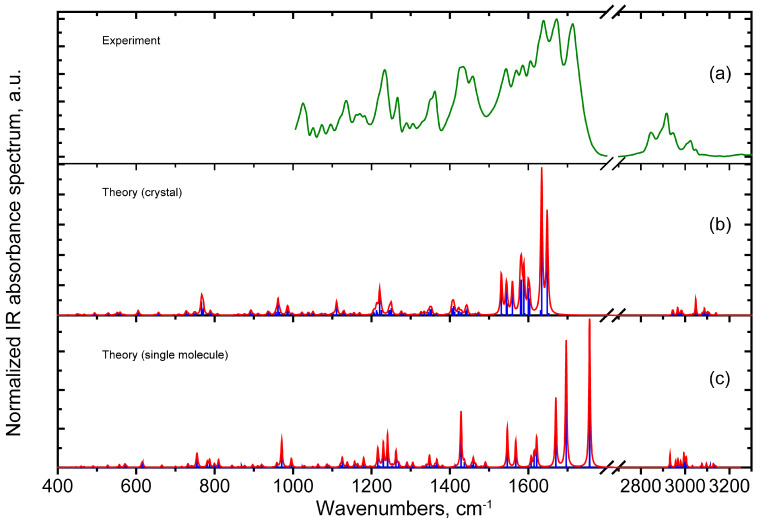
Normalized spectra: the experimental FTIR absorbance spectrum in the 1000–3300 cm^−1^ region (**a**), the theoretical IR absorbance spectrum in the 400–3300 cm^−1^ region simulated using the crystal calculations (**b**), and the single-molecule approach (**c**). In the case of the single-molecule calculations, the vibrational frequencies are scaled by a factor of 0.98.

Figure 3 demonstrates the obtained experimental FTIR spectra, as well as the theoretically simulated IR absorbance spectra. The interpretation of the observed bands in the region of 1000–1700 cm^−1^ is given in the Table 4. The spectrum in the regions 400–1000 and 2800–3100 cm^−1^ is discussed in the text.

In the obtained experimental spectrum, it is possible to distinguish a high-frequency region (2800–3100 cm^−1^), as well as a low-frequency region (below 1715 cm^−1^).

The presence of complex-shape wide bands is noted in the high-frequency region. The peaks in these regions are attributed to the stretching vibrations of the C-H bonds with different C atom hybridization states. Due to the significant anharmonicity of such vibrations [56], a detailed analysis of these vibrational modes is presented in comparison with the typical frequency ranges noted in Refs. [57,58].

The frequencies at the maximum points of the bands in the experimental spectrum can be divided into two groups, which belong either to the range of 2800–3000 cm^−1^ or 3000–3100 cm^−1^. The ν(C-H) vibrations of the bond with C atoms in sp^3^ hybridization are characteristic for the range of 2800–3000 cm^−1^. At the same time, the band with a maximum of about 2928 cm^−1^, interpreted as the ν_as_(CH_2_) mode, has the greatest IR intensity, while the band with a maximum of 2858 cm^−1^ belongs mainly to the ν_s_(CH_2_) mode. At the same time, the frequencies of individual ν(CH) modes for the single C-H bonds with the C atom in sp^3^ hybridization are predicted within the same range. Thus, the above-mentioned vibrational modes in the range of 2800–3000 cm^−1^ can be considered a characteristic manifestation of the individual structural features (rings IV and V) in the cytisine moiety. Vibrational modes in the range of 3000–3100 cm^−1^, which manifest themselves as features at about 3036 and 3060 cm^−1^, were attributed to vibrations of the type ν(=C-H), i.e., to the C-H bond stretching vibrations with C atoms in the sp^2^ hybridization state. In both calculations, the frequencies of the stretching vibrations are overestimated due to anharmonicity, but the ratio ν_sym_ (CH_2_) < ν_asym_ (CH_2_) < ν(=C-H) is maintained.

In the region below 1715 cm^−1^, the three most intense bands are observed in the experiment. In the calculation, these peaks correspond to vibrational modes with significant atomic displacements of the C=O bonds. Moreover, in both approaches, the ν(C=O) mode, localized in the coumarin moiety, has the highest frequency. This correlates with the fact that the O31 atom is located at a rather large distance from the H22 and H1 atoms (see Table 3). Further, the H15-O28, H16-O28, and H35-O28 contacts associated with the O28 atom belonging to the cytisine moiety lead to the reduced length of the intramolecular hydrogen bond. This correlates with the intermediate frequency of 1672 cm^−1^. Equally, the hydrogen bond O21-H3, in which one of the hydrogen atoms of the methyl group is turned towards the C=O bond, is the shortest one. It should be noted that in this mode, there is also a contribution from the stretching vibrations of the double bonds in the coumarin moiety, which is predicted by both types of calculations. In this case, the vibrational frequencies in the crystal calculation are predicted to be underestimated, which correlates with the above-mentioned overestimation of the lengths of the double bonds (including C=O). For the calculations within the single-molecule approach, the situation is the opposite: the frequency values are overestimated due to the lack of the environmental influence on these strongly polar bonds.

The spectral range of 1530–1620 cm^−1^ in the experimental spectrum is interpreted as predominantly consisting of ν(C=C) vibrations coupled with the ρ(CH) modes localized in the coumarin and cytisine moieties. In the range of 1400–1480 cm^−1^, a fairly wide band with a maximum of about 1431 cm^−1^ can be distinguished. According to the calculations, a characteristic feature of the modes in this range is the contribution of the ν(C-N) vibrations localized in the transition region, as well as in the cytisine moiety. Also, on the high-frequency side of this spectral feature is a peak of about 1460 cm^−1^, resulting mainly from δ(CH_2_) vibrations.

The absorption in the range of 1325–1375 cm^−1^ is related to modes with delocalized atomic displacements of various types in both parts of the complex. In the cytisine moiety, these are wagging-type deformations of the CH_2_ groups. In the coumarin moiety, these are mixtures of ν(C-C) and δ(HCC) vibrations.

In the range of 1200–1275 cm^−1^ in the FTIR spectrum, there are two significant bands with clearly asymmetric contours, which are drawn towards lower wave numbers. The mode associated with the peak at 1267 cm^−1^ is significantly localized in the cytisine part. At the same time, a more intense peak at 1233 cm^−1^ can be attributed to the ν(C-O) vibration in the coumarin moiety. The high polarity of this vibration makes it very active in the IR absorption spectrum.

The FTIR spectrum in the range of 1000–1200 cm^−1^ includes mainly the ν(C-C), ν(C-N), ν(C-O), and δ(CCH) contributions. A summary of the experimentally observed IR absorbance peaks and their attributions is given in Table 4.

In the range below 1000 cm^−1^, the calculation predicts an intense mode in the range of 960–970 cm^−1^, mainly associated with the ν(C-O) vibration in the coumarin moiety. It has high polarity and is very active in the IR absorption spectrum. In the region of 800–900 cm^−1^, the calculation predicts the presence of a large number of modes related to the ν(C-C) vibrations. Due to the low polarity of the bonds, this region is weakly manifested in the absorption spectrum. In the range of 400–800 cm^−1^, significant contributions from two types of vibrations are expected: from the deformation vibrations of the core and from the out-of-plane vibrations of the C-H bonds, which have relatively low polarity for the most part. Both types of calculations predict the greatest absorption activity in this range of 715–825 cm^−1^. These spectral features are related to the modes with complex atomic displacements involving out-of-plane vibrations of the oxygen atoms in the coumarin ring, as well as the out-of-plane vibrations of the C-H bonds.

### 3.3. Electronic Properties

The electronic structure of a crystal determines the scope of its possible applications. In this regard, the electronic band structure, as well as the charge distributions and populations according to Mulliken and Hirshfeld analyses, of the coumarin–cytisine complex crystal were analyzed.

The analysis of the electron distribution over the atoms based on the calculated charges according to the Mulliken and Hirshfeld analyses showed the following general trends:

(1) The charges on all H atoms are positive: from 0.25 e to 0.30 e according to the Mulliken analysis (from 0.03 e to 0.05 e according to the Hirshfeld analysis).
(2) The charges on all N atoms are negative: from −0.33 e to −0.29 e according to the Mulliken analysis (from −0.04 e to 0.00 e according to the Hirshfeld analysis).
(3) The charges on all O atoms are negative. The charges of the O atoms forming the C=O bonds lie in a range from −0.63 e to −0.57 e according to the Mulliken analysis (from −0.24 e to −0.20 e according to the Hirshfeld analysis). The charges on the O atoms forming the C-O-C bridges are lower; they are −0.42 e according to the Mulliken analysis (−0.06 e according to the Hirshfeld analysis).
(4) The charges on the C atoms forming the C-H bonds are negative and lie in the range from −0.47 e to −0.18 e according to the Mulliken analysis (from −0.08 e to −0.00 e according to the Hirshfeld analysis).
(5) The charges on the C atoms forming the C-O and C-N bonds are positive and lie in the range from 0.17 e to 0.61 e according to the Mulliken analysis (from 0.06 e to 0.16 e according to the Hirshfeld analysis).
(6) The charges on the C atoms that form the CC bonds only are negative and range from −0.13 e to −0.05 e according to the Mulliken analysis (from −0.03 e to −0.01 e according to the Hirshfeld analysis).

Thus, it can be stated that in the molecule under study, all of the H atoms are donors—they give up part of their electron density to the other, more electronegative atoms. All heteroatoms (O and N) are acceptors—they accept part of the electron density from the other, less electronegative atoms.

The population of the C atoms depends on the environment: C atoms that participate in the formation of C-H bonds are acceptors; C atoms that participate in the formation of C-O and C-N bonds are donors; and C atoms that participate in the formation of the CC bonds only are weak acceptors. The corresponding distribution of the total electron density and the electron localization functions demonstrating the above-mentioned trends are shown in Figure S3.

**Figure 4:**
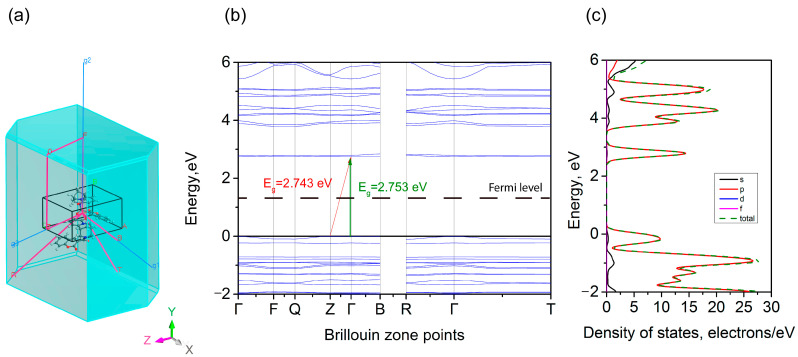
Brillouin zone points (**a**), electronic band structure (**b**), and partial density of states (**c**).

The set of special points in the Brillouin zone, the calculated electronic band structure, and the partial density of the electronic states are shown in Figure 4. The list of special k-points is given in Table S2. The calculated band structure exhibits the relatively gentle shape of the electronic states near the maximum of the valence band along the Z-Г-V path.

The top of the valence band is reached at point Z. It should be noted that the maxima at the Г and V points are only 9 and 3 meV lower.

The bottom of the conduction band is characterized by a rather narrow isolated split-off energy band. It is also characterized by rather gentle dependence along the Z-Г-V path. The minimum of the conduction band is reached at the Г point. At the same time, the minima at the Z and V points are predicted to be only 2 and 1 meV higher. From a formal point of view, it can be said that the system under study is an indirect band gap dielectric with an estimated band gap of 2.743 eV. The direct band gap, which occurs at the Г point, is estimated to be 2.752 eV. A comparison of the experimental and theoretical band gaps is discussed in the Supporting Information in Figure S4 and the corresponding text.

**Figure 5:**
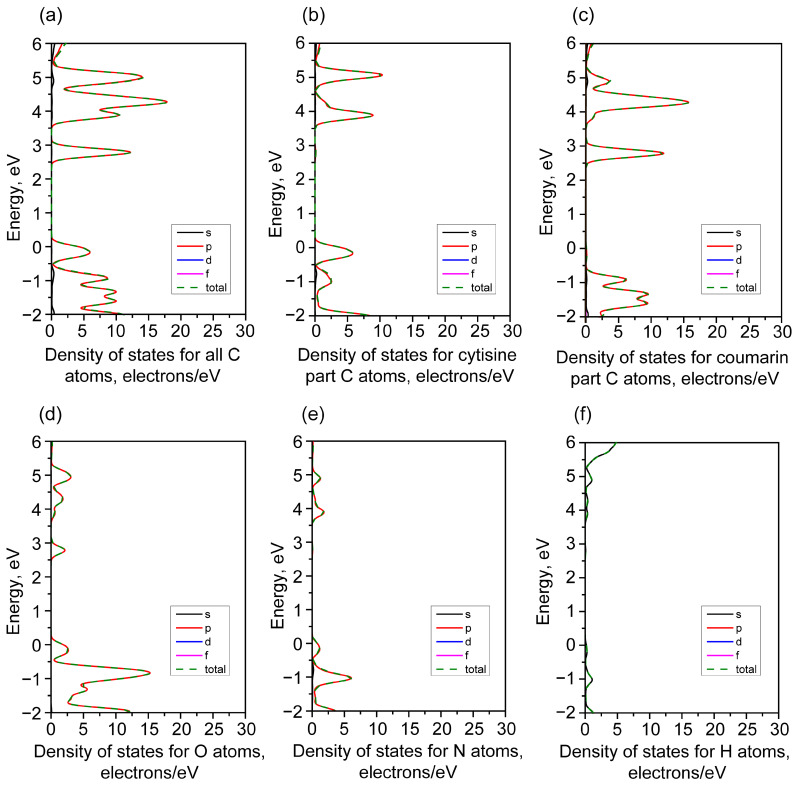
The density of the electronic states of all C atoms (**a**), cytisine part C atoms (**b**), coumarin part C atoms (**c**), O atoms (**d**), N atoms (**e**), and H atoms (**f**).

The partial atomic contributions to the density of the electronic states are shown in Figure 5. As can be seen, the main contribution to the lowest energy transition should be due to the electrons from the *p* orbitals.

In this case, the *p* electrons in the carbon atoms make an overwhelming contribution to the electronic states of the top of the valence band and the bottom of the conduction band. However, when analyzing the DOS distributions for the atoms in different parts of the complex—the cytisine and coumarin moieties—the following conclusions can be drawn. According to the calculation, the top of the valence band is formed mainly of the *p* electrons in the cytisine part, and the bottom of the conduction band is formed of the *p* electrons in the coumarin part. That is, electron excitation proceeds through electron transfer from the top of the valence band to the bottom of the conduction band (or in the opposite direction), which includes a spatial charge transfer between the cytisine and coumarin moieties.

## 4. Conclusions

In this work, a promising new compound with cytisine and coumarin parts was synthesized. According to the IUPAC classification, this complex molecule is N-(2-oxo-2H-chromen-3-carbonyl)cytisine. The structure of this compound, identified using XRD at room temperature, was found to correspond to the space group P2_1_. In the vibrational spectrum of this structure, calculated using density functional theory, one unstable mode localized in the ring of the cytisine unit was found. Distortion of the structure along the eigenvector of this mode led to the establishment of a stable crystal structure with P1 symmetry. It is shown that the energy difference between the symmetric unstable and asymmetric stable configurations is about 6.4 meV, which is much smaller than the thermal energy at room temperature.

The structural characteristics of the optimized stable structure (the bond lengths and valence angles) were calculated within the framework of two approaches (an ideal periodic crystal and a single molecule) and compared with the experimental data. It was found that the crystal calculation using the TS-GGA-PBE method provided overestimated values for the bond lengths but at the same time reproduced the value of the valence angles quite accurately. This led to underestimated values for the vibrational frequencies in the region of stretching vibrations of the double carbon bonds. At the same time, molecular calculation of the scaled frequencies using the B3LYP method with the 6-311G(2d,p) basis set gives close or slightly overestimated values for the vibrational mode frequencies. By comparing the calculated and experimental IR absorption spectra, assignments for the observed spectral peaks are proposed. The analysis of the atomic displacement localization in the modes corresponding to different peaks allowed us to divide the spectral lines into groups, which characterized the coumarin, cytisine, and intermediate units, as well as distinguishing the modes of a delocalized character. The high IR activity and the large frequency spread for the v(C=O) bond modes (1712, 1672, and 1638 cm^−1^) can be explained by the pronounced localized character of the corresponding atomic displacements. These modes can be considered markers of the corresponding structural units.

The theoretical study of the electronic zone structure showed that the highest energy state of the valence band exhibits weak dispersion in the Z-Г-B direction of the Brillouin zone. The valence zone maximum corresponds to the Z point. The minimum point of the conduction zone is localized near the Г point. From this point of view, the crystal can be considered to have an indirect forbidden zone equal to 2.743 eV. However, the direct gap at the Г point has a rather close energy value. The study of the partial densities shows that the lowest energy transition is associated with the charge transfer between the cytisine and coumarin parts of the molecule.

## Figures and Tables

**Table 1:** The unit cell parameters.

	Unit Cell Vector Length, Å			Unit Cell Angles, Å		
	a	b	c	α	β	γ
Experiment	9.3350(11)	7.6154(7)	12.7755(15)	90	102.794(4)	90
Theory *	9.3048	7.4090	12.7916	88.716	102.972	89.440

* for stable crystal structure with P1 symmetry.

**Table 3:** Lengths (in Å) of the intramolecular contacts between the oxygen and hydrogen atoms.

Selected H-O Contact	Experimental Data for the Complex Crystal P2_1_ (This Work)	Experimental Data for the Cytisine Crystal [53] *	Theoretical Data for the Complex Crystal P1 Molecule1 (Molecule2)	Theoretical Data for a Single Molecule
H22-O31	2.755(3)	--	2.632 (2.568)	2.675
H1-O31	3.135(3)	--	3.218 (3.172)	2.783
H3-O21	2.334(3)	--	2.281 (2.301)	2.242
H4-O21	3.346(3)	--	3.545 (3.514)	3.437
H35-O28	2.608(4)	--	2.643 (2.647)	2.627
H15-O28	2.418(4)	2.47(5)	2.417 (2.378)	2.436
H16-O28	2.628(4)	2.57(6)	2.605 (2.658)	2.571

* For the experimental data, the uncertainties are given in brackets.

**Table 4:** Peak positions in the experimental FTIR spectrum compared with the calculated frequencies of the modes with their interpretation.

Peak Number	Experimental Peak Frequency, cm^−1^	Calculated Frequency in the Crystal Approach *, cm^−1^	Calculated Frequency for a Single Molecule **, cm^−1^	Interpretation
1	1712	1648	1756	v(C=O) in coumarin part
2	1672	1634	1696	v(C=O) in cytisine part
3	1638	1600	1670	v(C=O) in intermediate part coupled with C=C in coumarin part
4	1607	1589	1621	v(C=C) and δ(CCH) in coumarin part
5	1586	1582 1580	1614	v(C=C) and δ(CCH) in coumarin part
6	1570	1560	1607	v(C=C), v(C=O) and δ(CCH) in cytisine part
7	1543	1531	1546	v(C=C) and δ(CCH) in cytisine part
8	1459	1421–1427	1460	δ(CH_2_)
9	1431	1410	1428	v(CN) in intermediate part and v(CN) and δ(CCH) in cytisine part
10	1364	1348 1353	1367	v(C-C) and δ(CCH) in coumarin part, as well as wagging modes in CH_2_ groups in cytisine part
11	1309	1285	1305	τ(CH_2_) in cytisine part
12	1291	1275	1290	δ(CCC), δ(CCH), v(C-C) in coumarin part
13	1267	1244 1247 1251	1262	predominantly v(CN), δ(CCN), τ(CH_2_) in cytisine part
14	1233	1220 1212 1205	1241 1230 1216	τ(CH_2_), δ(CCH), v(C-C), v(C-N) in cytisine part and v(C-C), v(C-O) δ(CCH) in coumarin part
15	1186	1155 1169	1157 1164	v(C-C), δ(CCH) in cytisine and coumarin part
16	1173	1130	1138	v(C-C), δ(CCH) in cytisine and coumarin part
17	1136	1111	1126	v(C-C),v(C-N), δ(CCH) in cytisine part
18	1024	987	996	v(C-C) in cytisine part and v(C-O) in coumarin part

* Modes localized in two molecules have close frequencies and similar atomic displacements. Thus, only one of the coupled modes is listed. ** Given values are scaled by a factor of 0.98.

## Data Availability

The original contributions presented in this study are included in the article and Supplementary Materials. Further inquiries can be directed to the corresponding authors.

## Supplementary Materials

The following supporting information can be downloaded at https://www.mdpi.com/article/10.3390/ma18133153/s1. Table S1: Selected structural parameters for the coumarin and cytisine moieties according to the experimental data and theoretical predictions; Figure S1: The experimental absorbance spectrum of N-(2-oxo-2H-chromen-3-carbonyl)cytisine (a), the absorbance spectrum calculated using the crystalline approach with a 0.82 eV scissor operator and 0.2 eV smearing for the optimized structure of the crystal complex within the TS-GGA-PBE approach (b), and the TDDFT calculations of the singlet–singlet vertical transition absorbance spectrum of a single molecule calculated within the B3LYP approach with the 6-311G (2d,p) basis set (c); Figure S2: Atomic displacements in the vibrational mode with the imaginary frequency (17.72 i cm^−1^) for the P2_1_ crystal structure; Figure S3. The distribution of the total density with the plane section at 0.32b (a) and the electron localization function with the plane section at 0.26b (b). Table S2: The Brillouin zone points and their coordinates; Table S3. Calculated singlet–singlet vertical transitions with the highest oscillator strength in the 200–500 nm region; Figure S4. The Tauc plots in cases of direct forbidden (left) and indirect forbidden (right) band gap estimations; Figure S5. The selected molecular orbitals involved in the singlet–singlet transitions demonstrated in Table S3.
