# Supplementary material for: Investigation of N-(2-oxo-2H-chromen-3-carbonyl)cytisine’s Crystal Structure and Optical Properties
Source: Materials (Basel). 2025 Jul 3;18(13):3153. doi: 10.3390/ma18133153 (PMC12251168; doi:10.3390/ma18133153)
Supplement: Supplementary file 1 [file materials-18-03153-s001.zip › Kopbalina2025 SuppInfnew.pdf]

Supporting information

for

Investigation of N-(2-oxo-2H-chromen-3-carbonyl)cytisine's Crystal Structure and Optical Properties

Anarkul Kishkentayeva[1], Kymbat Kopbalina[2*], Zhanar Shaimerdenova[1], Elvira Shults[2], Yury Gatilov[2], Dmitrii Pankin[4,*], Mikhail Smirnov[5], Anastasia Povolotckaia[4], Dastan Turdybekov[6], Nurlan Mazhenov[6]

[1] School of Pharmacy, Karaganda Medical University, Karaganda 100012, Kazakhstan; e-mail: anar_kish@mail.ru
[2] Department of Physics and Nanotechnology, Buketov Karaganda University, Universitetskaya 28, Karaganda 100024, Kazakhstan; kymbatkargtu@gmail.com
[3] N.N. Vorozhtsov Novosibirsk Institute of Organic Chemistry, Siberian Branch of the Russian Academy of Sciences, 630090 Novosibirsk, Russian Federation, e-mail: schultz@nioch.nsc.ru
[4] Center for Optical and Laser Materials Research, St. Petersburg State University, Ulianovskaya 5,
198504 St. Petersburg, Russia; dmitrii.pankin@spbu.ru
[5] Faculty of Physics, St. Petersburg State University, Universitetskaya Nab. 7/9, 199034 St. Petersburg, Russia; m.smirnov@spbu.ru
[6] Department of Physics, Abylkas Saginov Karaganda Technical University, Nazarbayev 56, Karaganda 100027, Kazakhstan; turdas@mail.ru, mazhenov@mail.ru
* Correspondence: kymbatkargtu@gmail.com (K.K.), dmitrii.pankin@spbu.ru (D.P.)

Table S1. Selected structural parameters for coumarin and cytisine moieties according to experimental data and theoretical predictions.

**Structural parameter**	**Experimental data for complex crystal SG P2$_1$ (this work)**	**Experimental data for coumarin crystal** [54,55]*	**Theoretical data for complex crystal approach, SG P1 molecule1 (molecule2)**	**Theoretical data for single molecule approach**
Plane angle, °				
C34C38C40	119.9(4)	120.5(3)	120.06 (120.11)	120.47
C38C40C43	120.7(4)	120.3(3)	120.04 (120.08)	119.87
C40C43C39	121.1(5)	120.4(2)	121.12 (121.08)	120.84
C43C39C37	117.5(4)	118.8(3)	118.28 (118.26)	118.91
C39C37C34	122.8(3)	122.2(2)	121.80 (121.89)	121.37
C37C34C38	117.9(3)	117.8(2)	118.63 (118.53)	118.55
C20C26C34	121.0(3)	120.5(3)	120.58 (120.90)	122.00
C26C34C37	118.5(3)	118.1(2)	118.36 (118.22)	117.46
C34C37O32	120.3(3)	120.6(2)	120.70 (120.61)	121.07
C37O32C25	122.2(2)	122.1(2)	122.41 (122.61)	123.01
O32C25C20	117.0(3)	117.4(2)	116.79 (116.79)	116.36
C25C20C26	120.9(3)	121.2(2)	121.06 (120.73)	120.41
O32C25O31	116.9(3)	116.7(2)	116.37 (116.24)	117.32
C25C20C13	118.9(3)	--	120.22 (120.69)	120.23
C20C13O21	119.5 (3)	--	120.25 (120.00)	118.35
Dihedral angle, °				
C26C20C13O21	-64.9(4)	--	-61.08 (-58.76)	-48.13
C25C20C13O21	113.1(3)	--	116.63 (118.15)	123.46

C20C13N10C14	1.2(4)	--	-3.71 (-1.63)	-6.81
C20C13N10C2	-175.5(3)	--	-172.10 (-171.52)	-186.84

**Structural parameter**	**Experimental data for complex crystal SG $P2_1$ (this work)***	**Experimental data for cytisine crystal** [53]*	**Theoretical data for complex crystal approach SG P1 molecule1 (molecule2)**	**Theoretical data for single molecule approach**
Plane angle, °				
N17C23C27	115.8(4)	116.2(4)	115.56 (115.34)	115.05
C23C27C30	121.6(5)	121.2(5)	121.05 (121.05)	121.43
C27C30C24	120.5(6)	119.5(5)	120.68 (120.80)	120.63
C30C24C19	120.5(4)	121.4(5)	120.23 (120.15)	119.80
C24C19C17	119.3(3)	119.0(4)	119.40 (119.27)	120.10
C19C17C23	122.1(4)	122.7(4)	122.92 (123.20)	122.95
C5C11N17	114.9(4)	115.6(4)	114.89 (114.81)	114.9
C11N17C19	122.9(3)	123.1(4)	123.31 (122.92)	123.76
N17C19C12	118.4(3)	118.5(4)	118.93 (118.96)	118.76
C19C12C7	112.3(3)	110.3(4)	111.41 (112.06)	110.73
C12C7C5	106.3(3)	106.4(5)	107.06 (106.83)	106.55
C7C5C11	108.4(4)	110.4(4)	109.24 (108.69)	109.95
C2C5C7	110.4(3)	109.0(4)	109.81 (110.70)	109.67
C5C7C12	106.3(3)	106.4(4)	107.06 (106.83)	106.55
C7C12C14	109.8(3)	109.7(4)	109.37 (108.77)	109.98
C12C14N10	110.1(3)	110.2(4)	110.41 (110.51)	110.95
C14N10C2	114.2(3)	111.6(4)	114.36 (114.61)	115.33
N10C2C5	111.5(4)	109.6(4)	111.45 (111.63)	111.78
C14N10C13	125.3(3)	--	124.90 (124.57)	125.77
N10C13O21	122.9(3)	--	123.24 (123.11)	122.71
N10C13C20	117.7(3)	--	116.49 (116.85)	118.93
Dihedral angle, °				
C23N17C11C5	-172.1(4)	178.1(4)	-174.79 (-171.70)	-176.62
C24C19C12C7	-152.9(4)	-143.4(5)	-152.37 (-154.30)	-148.85
C5C11N17C19	14.6(6)	0.1(6)	10.84 (14.59)	7.50
C11N17C19C12	-6.7(5)	-1.6(7)	-5.59 (-6.93)	-5.22
N17C19C12C7	29.8(4)	34.3(6)	30.50 (29.41)	32.79
C19C12C7C5	-59.2(4)	-63.9(5)	-59.54 (-58.23)	-61.28
C12C7C5C11	66.0(4)	62.2(5)	64.45 (65.20)	63.62
C7C5C11N17	-44.4(5)	-31.1(6)	-40.41 (-43.83)	-37.13
C2C5C7C12	-60.3(4)	-61.5(5)	-60.64 (-60.27)	-61.39
C5C7C12C14	62.4(3)	60.1(5)	61.92 (62.84)	61.62
C7C12C14N10	-59.6(4)	-59.1(5)	-58.44 (-59.85)	-56.42
C12C14N10C2	54.1(4)	58.3(5)	53.99 (53.89)	51.12
C14N10C2C5	-52.6(4)	-59.8(5)	-53.02 (-50.85)	-51.51
N10C2C5C7	55.6(4)	61.5(6)	56.04 (53.98)	56.34
C2N10C13O21	2.4(5)	--	6.02 (6.09)	-7.81

*for experimental data the uncertainties are given in brackets

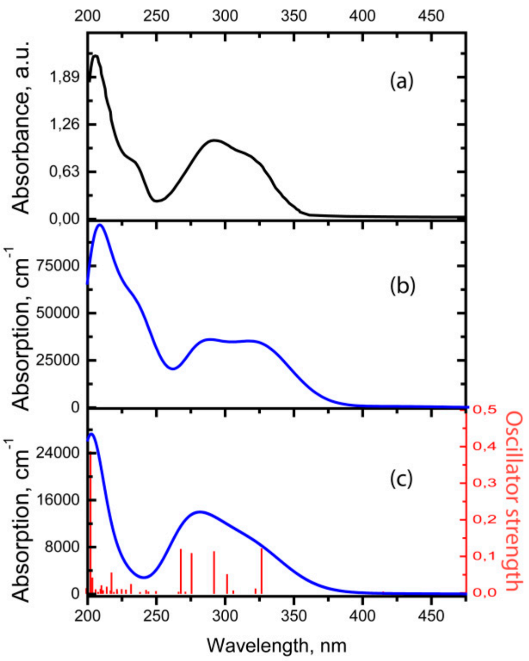

Figure S1. Experimental absorbance spectrum of N-(2-oxo-2H-chromen-3-carbonyl)cytisine (a), absorbance spectrum calculated in polycrystalline approach with scissor operator 0.82 eV and smearing 0.2 eV for the optimized structure of crystal complex within TS-GGA-PBE approach (b) and TDDFT calculations of singlet-singlet vertical transitions absorbance spectrum of single molecule calculated within B3LYP approach with 6-311G (2d,p) basis set.

In order to empirically define the scissor operator value the comparison was performed of the simulated absorbance spectrum in polycrystalline approach and experimental UV-Vis absorbance spectrum in the 200-475 nm wavelength region. The estimation of the scissor operator value was performed by enumerating the values so that the experimental (Figure S1a) and theoretical (Figure 1b) spectra coincide by peaks 206 and 292 nm as well as shoulders at 235 and 321 nm. For comparisonal reason the TDDFT calculated absorbance spectrum of singlet-singlet 40 lowest vertical transitions of single molecule calculated within B3LYP approach with 6-311G (2d,p) basis set is demonstrated at Figure S1c.

The single molecule optimized geometry within the B3LYP/6-311G(2d,p) approach

O	3.44092400	2.55829700	-1.56816200
C	2.31612100	-0.81100300	1.61148400
H	2.26929800	-0.83193700	2.70217100
O	0.17993900	-1.27922500	-2.41009100
C	1.06786400	-1.56550900	1.09099700
H	1.04268400	-2.56866100	1.53043700
H	0.17268800	-1.04214200	1.41284500
O	-1.57892900	-2.92068600	0.66493800
N	1.08401500	-1.65855600	-0.36031600

C	2.32161000	-2.17612400	-0.94001900
H	2.23347600	-2.09081800	-2.01999300
H	2.41362200	-3.23976100	-0.68462300
C	3.55357200	-1.42604900	-0.41510700
H	4.43222500	-1.92099600	-0.83776200
C	3.60730600	0.03494500	-0.86946800
H	4.64115300	0.38695400	-0.89481400
H	3.22779500	0.15262700	-1.88438700
N	2.85920400	0.98186900	-0.01323300
C	2.87042700	2.31837800	-0.51186600
C	2.18939700	3.28308600	0.31340900
H	2.17749400	4.29811200	-0.05907400
C	1.62449600	2.92947900	1.49776000
H	1.13267700	3.67800100	2.10960700
C	2.28772200	0.63806200	1.18195100
C	1.67554400	1.59098400	1.94887500
H	1.23491900	1.30391300	2.89364100
C	3.58882900	-1.49916300	1.11264200
H	3.61257400	-2.53896900	1.45031200
H	4.47790500	-1.00377200	1.51196400
O	-3.25253700	-1.46601300	0.69469000
C	-1.98244900	-1.85500800	0.28066600
C	-1.27074000	-0.92344300	-0.59412500
C	-1.86851300	0.21442600	-1.01280400
H	-1.34834700	0.85631800	-1.71515100
C	-3.17631400	0.58409300	-0.56285600
C	-3.82994300	1.76855200	-0.93867100
H	-3.32567900	2.45429400	-1.60958100
C	-5.09445000	2.05245800	-0.46111300
H	-5.59406100	2.96682800	-0.75466100
C	-5.73075900	1.15459500	0.40260800
H	-6.72294300	1.37794900	0.77547900
C	-5.10908900	-0.02196500	0.78778400
H	-5.58574000	-0.73080000	1.45212400
C	-3.83597700	-0.29945800	0.30254700
C	0.06043900	-1.30955700	-1.19381000

The optimized crystal structure within the TS-GGA-PBE approach

Element	Atom Number	Fractional coordinates of atoms u	v	w
H	1	0.043482	0.866297	0.121615
H	2	-0.103945	0.592971	0.119028
H	3	0.080735	0.537290	0.119743
H	4	-0.045869	0.466141	0.410158
H	5	-0.186785	0.549443	0.301301
H	6	-0.100031	0.798300	0.418099
H	7	0.140402	0.899319	0.475531
H	8	0.162354	0.663540	0.493232

H	9	0.620048	0.784175	0.421667
H	10	0.563064	0.809837	0.220380
H	11	0.299488	0.816689	0.118001
H	12	-0.163907	0.863561	0.217808
H	13	-0.023153	1.008633	0.283041
H	14	0.407859	0.350584	0.339148
H	15	0.657475	0.314900	0.303776
H	16	0.818452	0.223748	0.177798
H	17	0.705208	0.094691	0.003558
H	18	0.430392	0.075936	-0.057177
H	19	0.960897	0.372559	0.881085
H	20	1.107402	0.096642	0.873654
H	21	0.924124	0.045355	0.877022
H	22	1.028674	0.014789	0.576948
H	23	1.178448	0.071078	0.684091
H	24	1.095276	0.341992	0.580181
H	25	0.856546	0.469576	0.534935
H	26	0.830166	0.239220	0.502982
H	27	0.377898	0.335867	0.587391
H	28	0.440512	0.327174	0.788630
H	29	0.705533	0.321001	0.887617
H	30	1.165483	0.376039	0.781876
H	31	1.026795	0.535882	0.722286
H	32	0.588381	-0.122506	0.658512
H	33	0.340055	-0.165809	0.696197
H	34	0.185114	-0.264175	0.825536
H	35	0.305094	-0.401609	1.000859
H	36	0.580430	-0.421753	1.058666
C	1	0.053680	0.789639	0.197366
C	2	0.009278	0.593651	0.168043
C	3	-0.068205	0.555025	0.339075
C	4	-0.027392	0.751601	0.366904
C	5	0.130564	0.772108	0.432312
C	6	0.392751	0.779854	0.432174
C	7	0.507494	0.786802	0.373608
C	8	0.474666	0.799651	0.263627
C	9	0.213103	0.792910	0.257785
C	10	0.326693	0.803813	0.204998
C	11	-0.049010	0.867490	0.263857
C	12	0.146546	0.220967	0.117767
C	13	0.210521	0.289687	0.223247
C	14	0.359489	0.304014	0.258443
C	15	0.456368	0.255249	0.191166
C	16	0.611170	0.266734	0.223492
C	17	0.699127	0.213289	0.155033
C	18	0.634399	0.142371	0.054695
C	19	0.481850	0.131124	0.020228
C	20	0.394320	0.189964	0.088592
C	21	0.112686	0.340084	0.297795

C	22	0.948399	0.307225	0.803505
C	23	0.992600	0.106548	0.827494
C	24	1.058934	0.089149	0.651351
C	25	1.022964	0.291932	0.631342
C	26	0.864864	0.331766	0.569435
C	27	0.603769	0.335390	0.572949
C	28	0.491419	0.330105	0.633621
C	29	0.527333	0.325045	0.743728
C	30	0.787763	0.321131	0.745562
C	31	0.676197	0.321902	0.800489
C	32	1.050072	0.391170	0.737417
C	33	0.856081	-0.263627	0.878501
C	34	0.788893	-0.186450	0.773221
C	35	0.639192	-0.171898	0.739561
C	36	0.544877	-0.226440	0.808170
C	37	0.389582	-0.215479	0.777227
C	38	0.304790	-0.274179	0.847371
C	39	0.373199	-0.350224	0.948136
C	40	0.526203	-0.361959	0.981110
C	41	0.610410	-0.297768	0.910953
C	42	0.882966	-0.129403	0.696405
N	1	0.016869	0.482547	0.265325
N	2	0.245288	0.776644	0.368071
N	3	0.978695	0.008929	0.727386
N	4	0.752311	0.322762	0.635121
O	1	0.410881	0.776857	0.531406
O	2	0.123844	0.257341	0.384549
O	3	0.015994	0.197314	0.079171
O	4	0.244327	0.178187	0.053263
O	5	0.583023	0.349500	0.473687
O	6	0.867988	-0.202946	0.608681
O	7	0.987255	-0.291222	0.915798
O	8	0.760824	-0.310748	0.944612

The reduced Cif-file listing with the crystal geometry

```
data_anara1

_audit_creation_method            SHELXL-97
_chemical_name_systematic
;
 ?
;
_chemical_name_common             ?
_chemical_melting_point           ?
_chemical_formula_moiety          ?
_chemical_formula_sum
 'C21 H18 N2 O4'
_chemical_formula_weight          362.37

loop_
```

```
 _atom_type_symbol
 _atom_type_description
 _atom_type_scat_dispersion_real
 _atom_type_scat_dispersion_imag
 _atom_type_scat_source
 'C'  'C'   0.0033   0.0016
 'International Tables Vol C Tables 4.2.6.8 and 6.1.1.4'
 'H'  'H'   0.0000   0.0000
 'International Tables Vol C Tables 4.2.6.8 and 6.1.1.4'
 'N'  'N'   0.0061   0.0033
 'International Tables Vol C Tables 4.2.6.8 and 6.1.1.4'
 'O'  'O'   0.0106   0.0060
 'International Tables Vol C Tables 4.2.6.8 and 6.1.1.4'

_symmetry_cell_setting            ?
_symmetry_space_group_name_H-M    ?

loop_
 _symmetry_equiv_pos_as_xyz
 'x, y, z'
 '-x, y+1/2, -z'

_cell_length_a                    9.3350(11)
_cell_length_b                    7.6154(7)
_cell_length_c                    12.7755(15)
_cell_angle_alpha                 90.00
_cell_angle_beta                  102.794(4)
_cell_angle_gamma                 90.00
_cell_volume                      885.66(17)
_cell_formula_units_Z             2
_cell_measurement_temperature     296(2)
_cell_measurement_reflns_used     ?
_cell_measurement_theta_min       ?
_cell_measurement_theta_max       ?

_exptl_crystal_description        ?
_exptl_crystal_colour             ?
_exptl_crystal_size_max           0.69
_exptl_crystal_size_mid           0.15
_exptl_crystal_size_min           0.09
_exptl_crystal_density_meas       ?
_exptl_crystal_density_diffrn     1.359
_exptl_crystal_density_method     'not measured'
_exptl_crystal_F_000              380
_exptl_absorpt_coefficient_mu     0.095
_exptl_absorpt_correction_type    ?
_exptl_absorpt_correction_T_min   0.9372
_exptl_absorpt_correction_T_max   0.9915
_exptl_absorpt_process_details    ?

_exptl_special_details
;
 ?
;

_diffrn_ambient_temperature       296(2)
_diffrn_radiation_wavelength      0.71073
_diffrn_radiation_type            MoK\a
```

```
_diffrn_radiation_source          'fine-focus sealed tube'
_diffrn_radiation_monochromator   graphite
_diffrn_measurement_device_type   ?
_diffrn_measurement_method        ?
_diffrn_detector_area_resol_mean  ?
_diffrn_standards_number          ?
_diffrn_standards_interval_count  ?
_diffrn_standards_interval_time   ?
_diffrn_standards_decay_%         ?
_diffrn_reflns_number             14375
_diffrn_reflns_av_R_equivalents   0.0576
_diffrn_reflns_av_sigmaI/netI     0.0522
_diffrn_reflns_limit_h_min        -12
_diffrn_reflns_limit_h_max        12
_diffrn_reflns_limit_k_min        -9
_diffrn_reflns_limit_k_max        9
_diffrn_reflns_limit_l_min        -16
_diffrn_reflns_limit_l_max        16
_diffrn_reflns_theta_min          3.05
_diffrn_reflns_theta_max          27.50
_reflns_number_total              4057
_reflns_number_gt                 2859
_reflns_threshold_expression      >2sigma(I)

_computing_data_collection        ?
_computing_cell_refinement        ?
_computing_data_reduction         ?
_computing_structure_solution     'SHELXS-97 (Sheldrick, 1990)'
_computing_structure_refinement   'SHELXL-97 (Sheldrick, 1997)'
_computing_molecular_graphics     ?
_computing_publication_material   ?

_refine_special_details
;
 Refinement of F^2^ against ALL reflections.  The weighted R-factor wR
and
 goodness of fit S are based on F^2^, conventional R-factors R are
based
 on F, with F set to zero for negative F^2^. The threshold expression
of
 F^2^ > 2sigma(F^2^) is used only for calculating R-factors(gt) etc.
and is
 not relevant to the choice of reflections for refinement.  R-factors
based
 on F^2^ are statistically about twice as large as those based on F,
and R-
 factors based on ALL data will be even larger.
;

_refine_ls_structure_factor_coef  Fsqd
_refine_ls_matrix_type            full
_refine_ls_weighting_scheme       calc
_refine_ls_weighting_details
 'calc w=1/[\s^2^(Fo^2^)+(0.0592P)^2^+0.0129P] where
P=(Fo^2^+2Fc^2^)/3'
_atom_sites_solution_primary      direct
_atom_sites_solution_secondary    difmap
_atom_sites_solution_hydrogens    geom
```

```
_refine_ls_hydrogen_treatment     mixed
_refine_ls_extinction_method      none
_refine_ls_extinction_coef        ?
_refine_ls_abs_structure_details
 'Flack H D (1983), Acta Cryst. A39, 876-881'
_refine_ls_abs_structure_Flack    -0.4(12)
_refine_ls_number_reflns          4057
_refine_ls_number_parameters      244
_refine_ls_number_restraints      1
_refine_ls_R_factor_all           0.0733
_refine_ls_R_factor_gt            0.0448
_refine_ls_wR_factor_ref          0.1190
_refine_ls_wR_factor_gt           0.1034
_refine_ls_goodness_of_fit_ref    1.020
_refine_ls_restrained_S_all       1.020
_refine_ls_shift/su_max           0.000
_refine_ls_shift/su_mean          0.000

loop_
 _atom_site_label
 _atom_site_type_symbol
 _atom_site_fract_x
 _atom_site_fract_y
 _atom_site_fract_z
 _atom_site_U_iso_or_equiv
 _atom_site_adp_type
 _atom_site_occupancy
 _atom_site_symetry_multiplicity
 _atom_site_calc_flag
 _atom_site_refinement_flags
 _atom_site_disorder_assembly
 _atom_site_disorder_group
O28 O 0.3961(3) 0.8224(6) 0.53014(17) 0.1473(13) Uani 1 1 d . . .
C12 C 0.0598(3) 0.7909(3) 0.19579(16) 0.0515(5) Uani 1 1 d . . .
H18 H 0.0505 0.8559 0.1284 0.062 Uiso 1 1 calc R . .
O21 O 0.1211(2) 0.2796(2) 0.38194(13) 0.0755(5) Uani 1 1 d . . .
C14 C 0.0238(2) 0.5979(3) 0.16918(15) 0.0472(5) Uani 1 1 d . . .
H1 H -0.0741 0.5884 0.1238 0.057 Uiso 1 1 calc R . .
H22 H 0.0930 0.5502 0.1302 0.057 Uiso 1 1 calc R . .
O31 O 0.02117(15) 0.2032(2) 0.08541(11) 0.0520(4) Uani 1 1 d . . .
N10 N 0.03147(18) 0.4986(2) 0.26705(13) 0.0439(4) Uani 1 1 d . . .
C2 C -0.0590(3) 0.5693(3) 0.3368(2) 0.0602(6) Uani 1 1 d . . .
H3 H -0.0397 0.5044 0.4038 0.072 Uiso 1 1 calc R . .
H4 H -0.1619 0.5542 0.3027 0.072 Uiso 1 1 calc R . .
C5 C -0.0278(3) 0.7633(3) 0.3602(2) 0.0613(6) Uani 1 1 d . . .
H6 H -0.0998 0.8067 0.3994 0.074 Uiso 1 1 calc R . .
C11 C 0.1228(3) 0.8023(5) 0.42690(18) 0.0733(7) Uani 1 1 d . . .
H16 H 0.1208 0.9169 0.4598 0.088 Uiso 1 1 calc R . .
H15 H 0.1458 0.7165 0.4843 0.088 Uiso 1 1 calc R . .
N17 N 0.2412(2) 0.8009(3) 0.36750(14) 0.0581(5) Uani 1 1 d . . .
C23 C 0.3834(3) 0.8146(6) 0.4320(2) 0.0940(10) Uani 1 1 d . . .
C27 C 0.4991(4) 0.8160(6) 0.3768(4) 0.1089(12) Uani 1 1 d . . .
H35 H 0.5957 0.8158 0.4160 0.131 Uiso 1 1 calc R . .
C30 C 0.4731(4) 0.8177(5) 0.2703(3) 0.0929(10) Uani 1 1 d . . .
H36 H 0.5512 0.8212 0.2361 0.112 Uiso 1 1 calc R . .
C19 C 0.2151(2) 0.8050(3) 0.25807(16) 0.0476(5) Uani 1 1 d . . .
C24 C 0.3302(3) 0.8145(3) 0.2097(2) 0.0663(7) Uani 1 1 d . . .
H29 H 0.3132 0.8188 0.1352 0.080 Uiso 1 1 calc R . .
```

```
C7 C -0.0484(3) 0.8663(3) 0.2563(2) 0.0659(7) Uani 1 1 d . . .
H8 H -0.1482 0.8536 0.2146 0.079 Uiso 1 1 calc R . .
H9 H -0.0288 0.9899 0.2711 0.079 Uiso 1 1 calc R . .
O32 O 0.24437(15) 0.17707(18) 0.05740(10) 0.0461(4) Uani 1 1 d . . .
C25 C 0.1504(2) 0.2259(2) 0.12127(14) 0.0374(4) Uani 1 1 d . . .
C20 C 0.2160(2) 0.3008(2) 0.22526(14) 0.0364(4) Uani 1 1 d . . .
C26 C 0.3618(2) 0.3139(3) 0.25846(15) 0.0430(4) Uani 1 1 d . . .
H33 H 0.4017 0.3592 0.3263 0.052 Uiso 1 1 calc R . .
C34 C 0.4572(2) 0.2596(3) 0.19149(17) 0.0436(5) Uani 1 1 d . . .
C38 C 0.6102(2) 0.2711(3) 0.2215(2) 0.0600(6) Uani 1 1 d . . .
H41 H 0.6550 0.3179 0.2880 0.072 Uiso 1 1 calc R . .
C40 C 0.6942(3) 0.2135(4) 0.1529(3) 0.0681(7) Uani 1 1 d . . .
H44 H 0.7959 0.2232 0.1728 0.082 Uiso 1 1 calc R . .
C43 C 0.6299(3) 0.1418(3) 0.0554(3) 0.0705(8) Uani 1 1 d . . .
H45 H 0.6889 0.1014 0.0106 0.085 Uiso 1 1 calc R . .
C39 C 0.4787(3) 0.1284(3) 0.0226(2) 0.0590(6) Uani 1 1 d . . .
H42 H 0.4348 0.0798 -0.0435 0.071 Uiso 1 1 calc R . .
C37 C 0.3956(2) 0.1897(2) 0.09173(16) 0.0424(5) Uani 1 1 d . . .
C13 C 0.1172(2) 0.3585(2) 0.29733(14) 0.0407(5) Uani 1 1 d . . .

loop_
 _atom_site_aniso_label
 _atom_site_aniso_U_11
 _atom_site_aniso_U_22
 _atom_site_aniso_U_33
 _atom_site_aniso_U_23
 _atom_site_aniso_U_13
 _atom_site_aniso_U_12
O28 0.1034(18) 0.264(4) 0.0558(12) -0.020(2) -0.0230(12) -0.034(2)
C12 0.0714(14) 0.0417(10) 0.0375(10) 0.0045(9) 0.0032(10) 0.0048(10)
O21 0.1106(15) 0.0748(11) 0.0513(9) 0.0259(9) 0.0396(10) 0.0265(10)
C14 0.0559(13) 0.0469(11) 0.0334(11) 0.0051(9) -0.0014(9) 0.0015(9)
O31 0.0382(8) 0.0664(9) 0.0483(8) -0.0103(7) 0.0032(6) -0.0077(7)
N10 0.0498(10) 0.0447(9) 0.0398(9) 0.0027(8) 0.0155(8) 0.0025(8)
C2 0.0535(14) 0.0650(14) 0.0695(16) 0.0019(13) 0.0294(12) 0.0048(11)
C5 0.0601(15) 0.0647(14) 0.0664(15) -0.0120(12) 0.0300(13) 0.0054(12)
C11 0.0801(18) 0.1031(19) 0.0407(12) -0.0157(14) 0.0223(12) -
0.0075(16)
N17 0.0523(11) 0.0832(13) 0.0378(9) -0.0058(10) 0.0080(8) -0.0099(10)
C23 0.0680(19) 0.141(3) 0.0629(17) -0.010(2) -0.0065(15) -0.024(2)
C27 0.0522(18) 0.146(3) 0.124(3) -0.009(3) 0.0108(19) -0.021(2)
C30 0.078(2) 0.090(2) 0.125(3) -0.005(2) 0.054(2) -0.0213(17)
C19 0.0624(14) 0.0449(10) 0.0371(10) -0.0018(9) 0.0145(9) -0.0044(10)
C24 0.084(2) 0.0579(14) 0.0672(15) -0.0029(13) 0.0381(15) -0.0114(13)
C7 0.0633(16) 0.0507(12) 0.0789(18) -0.0038(12) 0.0055(13) 0.0097(11)
O32 0.0404(8) 0.0611(9) 0.0365(7) -0.0095(7) 0.0077(6) -0.0017(6)
C25 0.0364(11) 0.0420(10) 0.0333(9) -0.0006(8) 0.0068(8) -0.0027(8)
C20 0.0383(10) 0.0388(9) 0.0314(9) 0.0027(8) 0.0064(8) 0.0008(8)
C26 0.0429(11) 0.0486(10) 0.0337(9) 0.0008(9) 0.0001(8) -0.0037(9)
C34 0.0342(10) 0.0475(10) 0.0486(11) 0.0064(9) 0.0077(9) -0.0026(8)
C38 0.0377(12) 0.0670(15) 0.0718(15) 0.0070(13) 0.0046(11) -0.0017(10)
C40 0.0388(13) 0.0670(15) 0.101(2) 0.0201(16) 0.0210(14) 0.0044(12)
C43 0.0625(17) 0.0653(15) 0.098(2) 0.0151(15) 0.0493(16) 0.0154(13)
C39 0.0620(15) 0.0587(13) 0.0634(15) 0.0002(12) 0.0292(12) 0.0018(12)
C37 0.0391(11) 0.0425(10) 0.0475(11) 0.0047(9) 0.0136(9) 0.0003(9)
C13 0.0474(12) 0.0461(10) 0.0290(9) 0.0052(8) 0.0093(8) -0.0011(9)
```

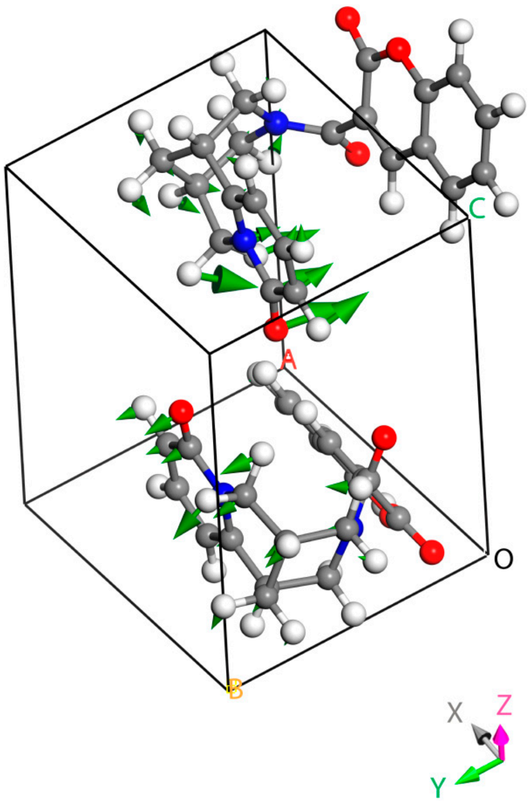

Figure S2. Atomic displacements in the vibrational mode with imaginary frequency(17.72i cm^{-1}) for the crystal with SG $P2_1$.

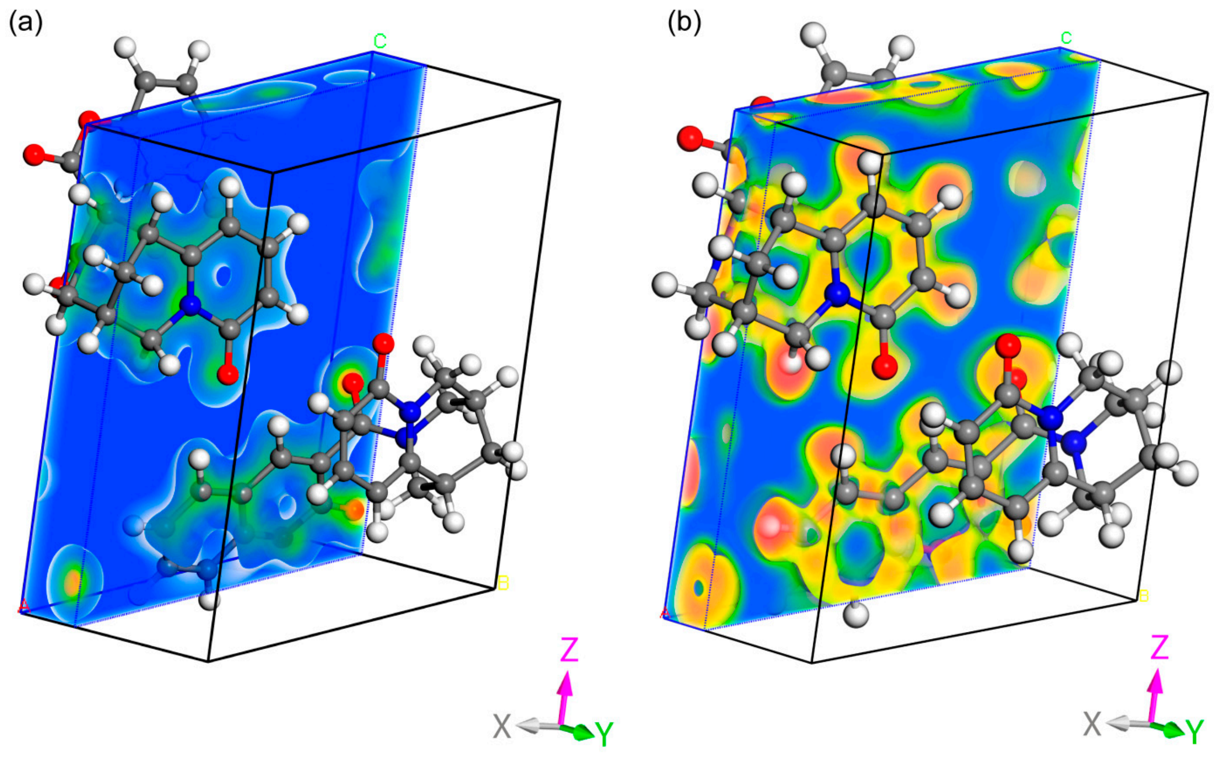

Figure S3. The distribution of total density with plane section at 0.32**b** (a) and distribution of electron localization function with plane section at 0.26**b** (b).

Table S2. Brillouin zone point and their coordinates.

Brillouin zone point	Coordinate
Γ	0 0 0
F	0 0.5 0
Q	0 0.5 0.5
Z	0 0 0.5
B	0.5 0 0
R	-0.5 -0.5 0.5
T	0 -0.5 0.5

<u>Interpretation of spectra absorption and correlation with electronic structure</u>

For clarity it was chosen the obtained results for the single molecules approach and discussed below. According to this calculations it is predicted the HOMO-LUMO transition about 415 nm with very low oscillator strength. The similar situation is in the case of crystal. Much higher oscillator strengths have transitions with the wavelengths in the region 250-330 nm (see Table S3). This agrees with the experimental spectra at Figure S1. From this point of view the band gaps were estimated on the Tauc plot approach with direct forbidden and indirect forbidden cases. It was obtained 2.787 and 3.021 eV band gap values correspondingly (see Figure S1). They are quite close to the theoretically predicted 2.753 and 2.743 eV correspondingly.

For experimentally observed broad band in the region 250-350 nm in Figure S1 the three transitions are predicted by theory. It is the transitions to the excited states 6,7,8. Their oscillator strength is predicted to be close.

Table S3. Calculated singlet-singlet vertical transitions with highest oscillators strength in the 200-500 nm region.

**Transition №**	**Excited state №**	**Orbitals with >10% Contribution (Percent)**	**Oscillator Strength**	**Wavelength, nm (Energy, eV)**
1	2	94 -> 96 (63)<br>90 -> 96 (12)	0.1196	326.72 (3.7948)
2	6	95 -> 97 (94)	0.1119	292.32 (4.2413)
3	7	89 -> 96 (39)<br>90 -> 96 (40)	0.1068	276.05 (4.4914)
4	9	89 -> 96 (43)<br>90 -> 96 (38)	0,1181	268.27 (4.6216)
5	30	91 -> 98 (43)<br>86 -> 96 (24)<br>90 -> 98 (12)	0,3851	202.92 (6.1099)

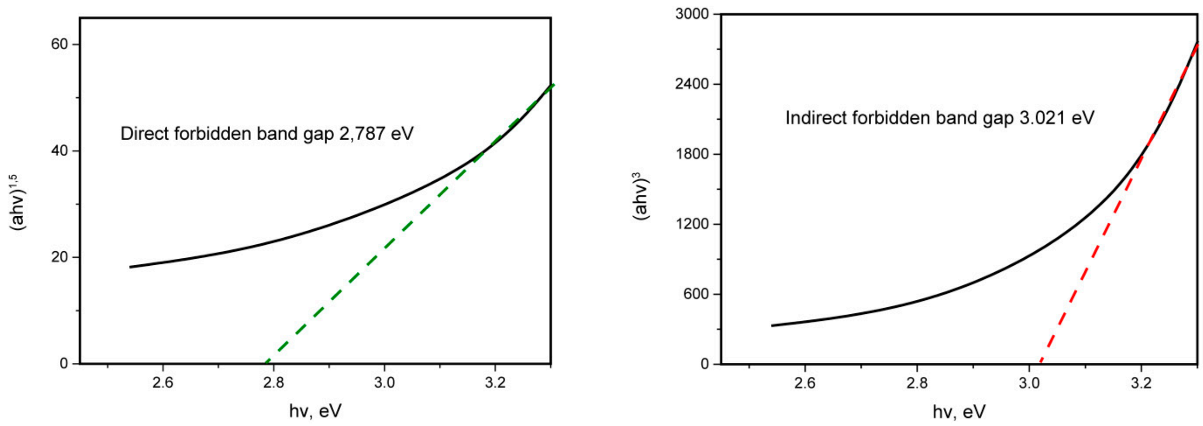

Figure S4. The Tauc plots in case of direct forbidden (left) and indirect forbidden (right) band gaps estimations.

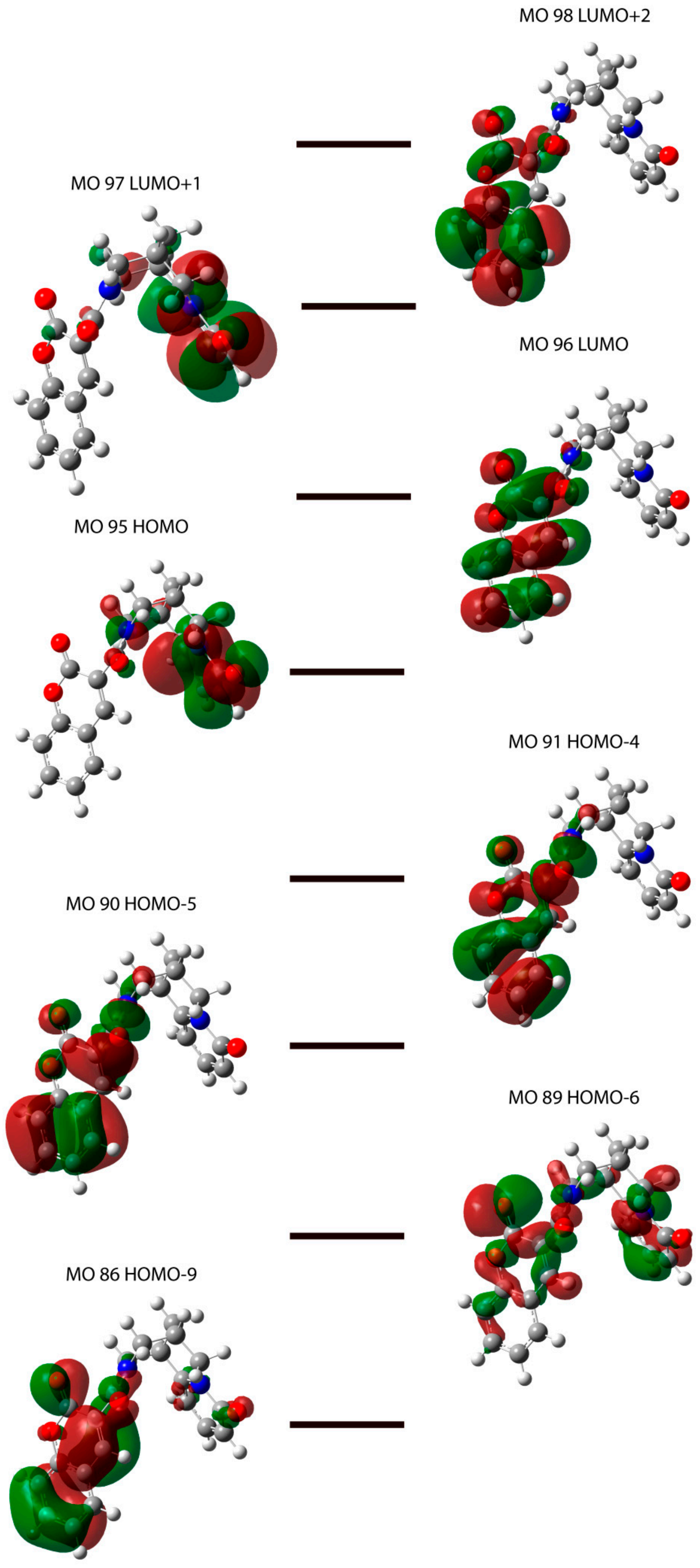

Figure S5. The selected molecular orbitals involved in singlet-singlet transitions demonstrated in Table S3.

References:

53. Freer, A.A.; Robins, D.J.; Sheldrake, G.N. Structures of (−)-Cytisine and (−)-N-Methylcytisine: Tricyclic Quinolizidine Alkaloids. *Acta Crystallogr. C* **1987**, *43*, 1119–1122. https://doi.org/10.1107/S0108270187092813.
54. Myasnikova, R.M.; Davydova, T.S.; Simonov, V.I. Crystal-Structure of Coumarin. *Kristallografiya* **1973**, *18*, 720–724.
55. Waddell, P.G.; Probert, M.R.; Johnson, N.T. *CCDC 2360262: Experimental Crystal Structure Determination*; Cambridge Crystallographic Data Centre: Cambridge, UK, 2024.
